# Native Valve Legionella Endocarditis in a 74-Year-Old Male: A Case Report and Review

**DOI:** 10.7759/cureus.71870

**Published:** 2024-10-19

**Authors:** Angelina A Porter, Anthony Abro, Nisha Desai, Habeeb Yazdani

**Affiliations:** 1 Internal Medicine, Ascension Health, Warren, USA; 2 Internal Medicine, Ascension St. John Hospital, Warren, USA

**Keywords:** blood culture-negative endocarditis, extrapulmonary legionella, infective endocarditis, legionella endocarditis, legionella infection, native valve endocarditis

## Abstract

Legionella is a bacterium that primarily causes respiratory infections. Rarely, it can result in extrapulmonary infections like endocarditis. We present a case of native valve endocarditis caused by Legionella in a 74-year-old male. Our patient initially presented with fever, fatigue, confusion, moderate cough, and dysuria. Subsequent presentation included atrial fibrillation and pulmonary infiltrates on chest X-ray. His clinical sequelae and positive urine Legionella antigen prompted treatment with antibiotics and cardiac medication. Echocardiograms revealed vegetation on the mitral valve leaflet suggestive of endocarditis. The patient improved after appropriate antibiotic and anti-arrhythmic therapy. In presenting this case and literature review, we emphasize the importance of considering atypical pathogens in common clinical presentations.

## Introduction

Legionella is a gram-negative bacterium commonly found in water sources, such as cooling towers, hot tubs, and plumbing systems. It is most often associated with pulmonary infections, including Pontiac fever, a mild, flu-like illness, and Legionnaires' disease, a severe form of pneumonia. Extrapulmonary infections (those outside the lungs), like infective endocarditis, are much less common. In fact, the incidence of Legionella causing infective endocarditis is less than 1% of all cases [1]. Endocarditis refers to an infection of the inner lining of the heart chambers and valves, known as the endocardium. While prosthetic (artificial) valves are most at risk of infection, it is less common for native (natural) valves to be involved [2].

In this report, we discuss a rare case of native valve endocarditis caused by Legionella, which presented unusually with arrhythmia (irregular heart rhythm). We also provide a review of reported cases of Legionella endocarditis to offer insights into this uncommon clinical presentation.

## Case presentation

**Figure 1:**
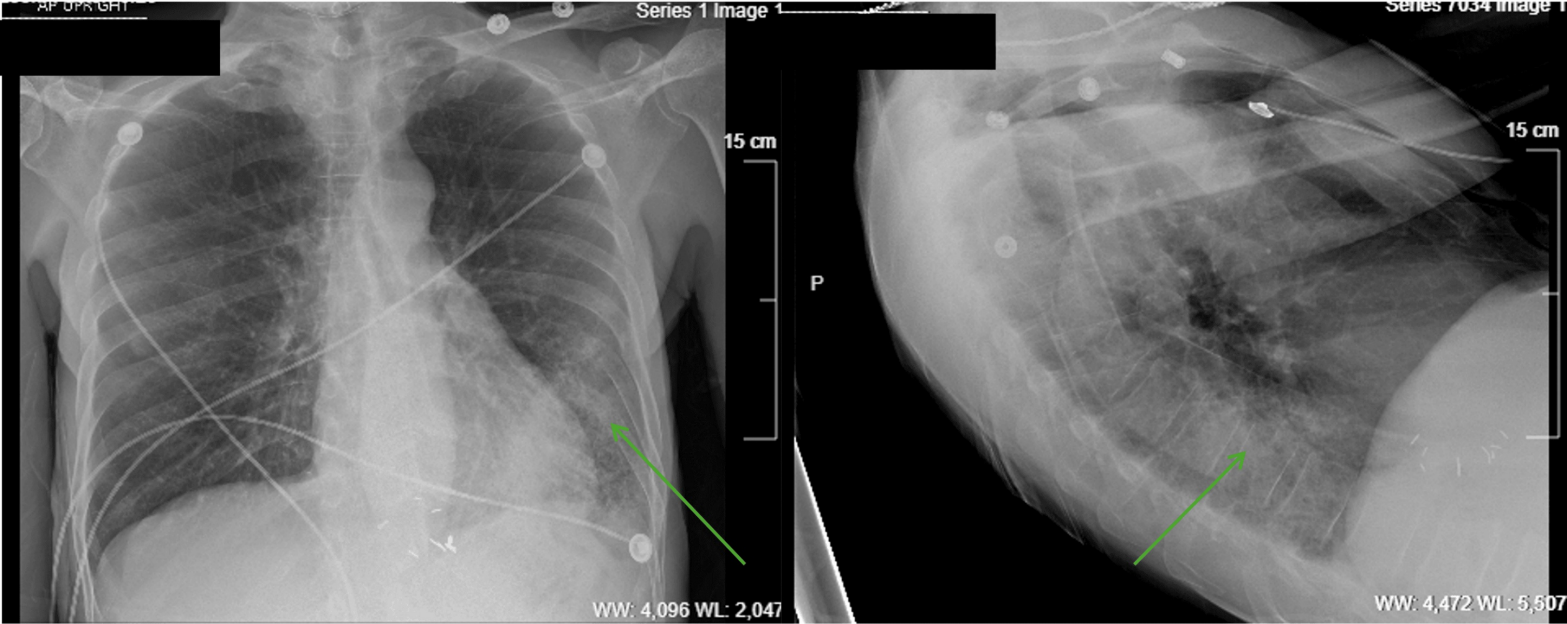
Two-view chest radiograph on day 0 exhibiting lower lobe acute process (green arrows).

A 74-year-old Black American male with a past medical history of uncontrolled hypertension, uncontrolled type 2 diabetes, chronic alcoholism, and chronic tobacco use presented to the emergency room following a syncopal episode. He was found on the ground by a bystander and did not recall the events prior to it. At the time, he admitted to fever, fatigue, and dysuria. Physical examination revealed a lethargic male with tachycardia and a fever of 102.8° Fahrenheit, in addition to bilateral rhonchi and decreased breath sounds at the lung bases. Laboratory results included leukocytosis (23.3 K/mcL), mild hyponatremia (127 mmol/L), lactic acidemia (2.3 mmol/L), and positive urinalysis with the growth of Citrobacter koseri on urine culture (Table 1). Blood cultures and COVID-19 polymerase chain reaction (PCR) were negative. Radiographic imaging revealed bilateral lower lobe airspace disease indicative of an acute process (Figure 1). He was admitted for suspected pneumonia and urinary sepsis and was started on cefepime and azithromycin.

**Table 1:** Patient's laboratory values on admission. K/mcL = cells per microliter multiplied by 1,000; mmol/L = millimoles per liter.

Laboratory markers	Patient values	Reference range
White blood cells (K/mcL)	23.3	4-11
Sodium (mmol/L)	127	135-145
Lactic acid (mmol/L)	2.3	0.5-2.0
Urine culture	Positive for growth	No growth

**Figure 2:**
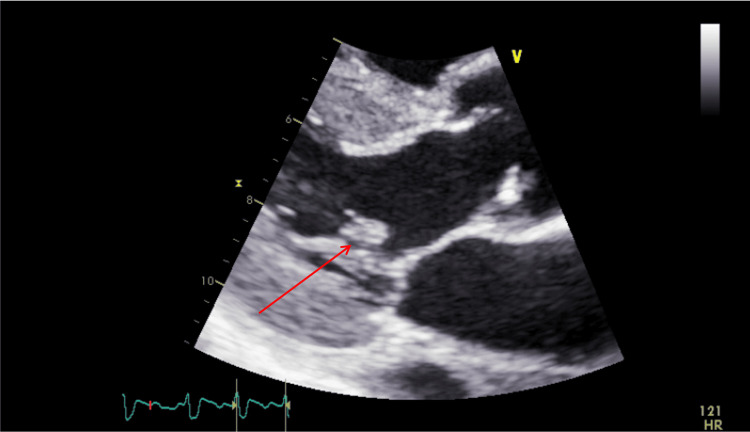
Mitral valve vegetation (red arrow) seen on echocardiogram on day three.

**Figure 3:**
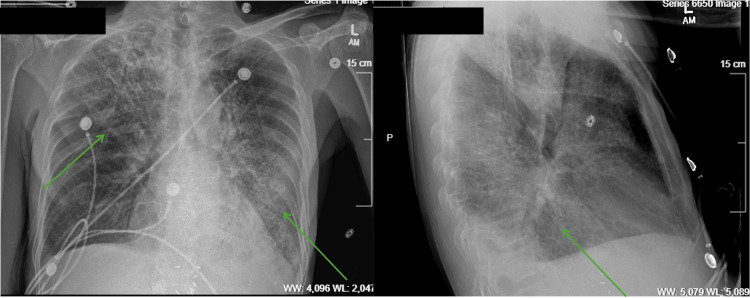
Two-view chest radiograph on day four exhibiting worsening bilateral infiltrate with ground-glass appearance (green arrows).

The patient’s condition continued to deteriorate after admission. He developed atrial fibrillation on day two and was resuscitated with adenosine. Following the cardiac event, amiodarone and heparin were added to his treatment regimen. An echocardiogram was performed on day three, showing vegetation on the anterior mitral valve leaflet measuring approximately 2.5 x 3.3 mm, in addition to an echo-dense mobile structure that was likely calcification of the chordae tendineae (Figure 2). These findings confirmed the diagnosis of infective endocarditis. Subsequent radiographic testing showed worsening bilateral lower lobe infiltrates (Figure 3). At this time, the patient required 6 L of oxygen via nasal cannula to saturate 97%. His care team was informed of his recent travel to Jamaica, prompting Legionella antigen testing, which resulted positive. Cefepime was discontinued and ciprofloxacin was added in its place for appropriate coverage.

**Figure 4:**
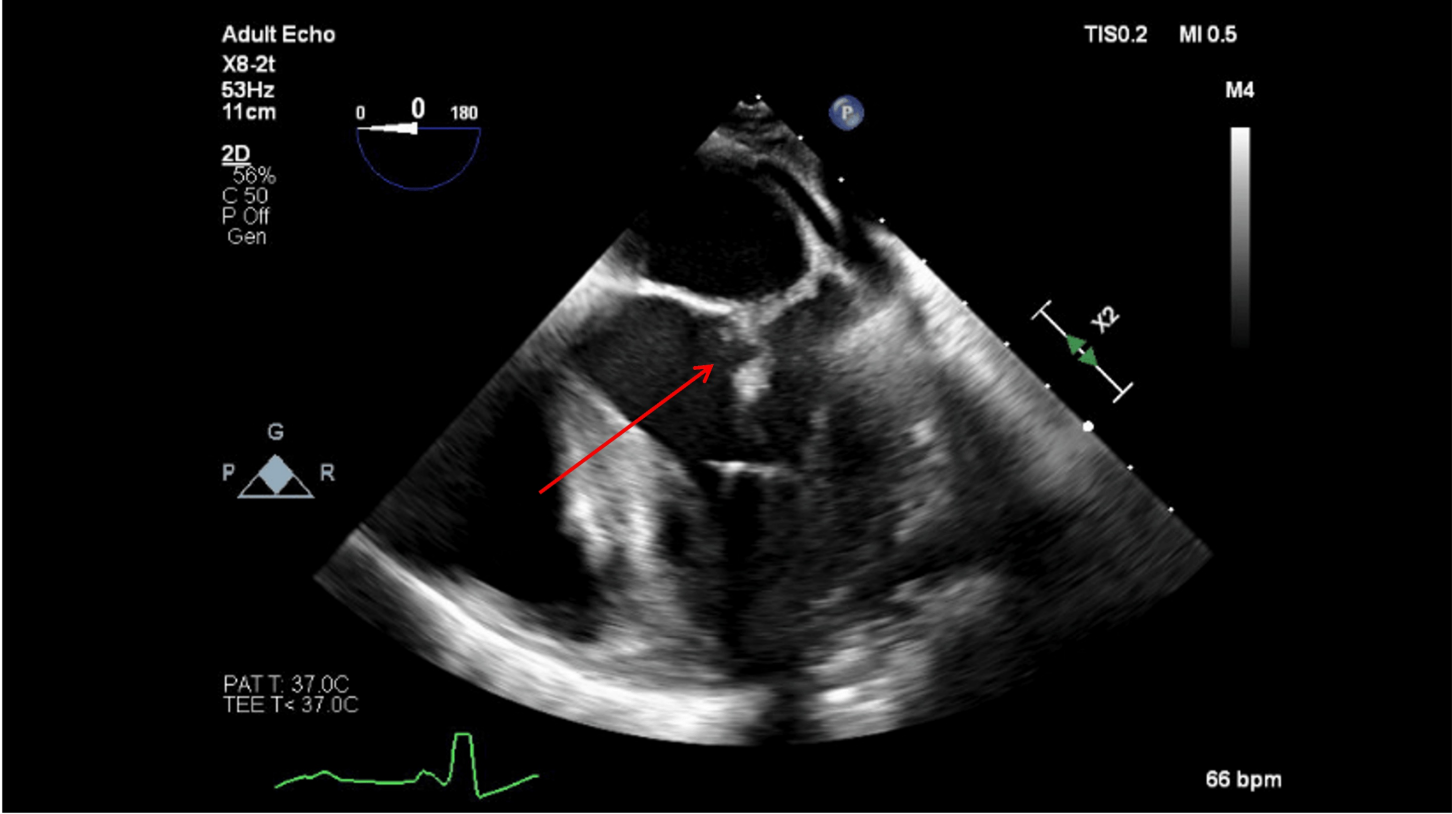
Mitral valve vegetation still visible on day four via transesophageal echocardiogram (red arrow).

By day five, the patient’s fever and leukocytosis were improving, but he was still tachypneic and required 10 L of oxygen via nasal cannula to saturate 95%. A transesophageal echocardiogram confirmed the presence of vegetation on the mitral valve leaflet (Figure 4).

The patient was discharged on day eight with instructions to continue cardiac medications in addition to a six-week course of azithromycin, rather than levofloxacin due to interactions with amiodarone. He was instructed to complete a follow-up chest X-ray eight weeks after discharge; however, it is unknown if the patient followed up with his primary care provider or cardiologist after discharge. He returned to the emergency department eight months later for generalized weakness. A two-view chest X-ray was completed at the time and there was no indication of acute processes.

## Discussion

Our methods for the literature review included searching for key phrases, including “Legionella endocarditis,” “extrapulmonary Legionella,” and “infective endocarditis” on PubMed. The number of total reported cases varies by publication, and we were able to find 24 reported cases going back as far as 1984. In this report, we review 16 of the 24 cases of Legionella endocarditis found in the literature, excluding eight due to insufficient data reported - they did not include certain patient demographics and co-morbidities that we were looking to evaluate, such as age, sex, reason for admission, and prognosis. The findings are summarized in Table 2. We included patient demographics, comorbidities, reason for admission, the valve affected and type of valve, diagnostic testing, treatment, and prognosis after treatment. Of the 16 cases, 10 patients were male and six were female, ranging from ages 28 to 80 years. Only five occurred on native valves, while the other 11 occurred on prosthetic valves (nine bioprosthetic and two mechanical prosthetic valves). The most common symptom was fever, reported in nine out of 16 cases. The most affected valve was the aortic valve (13), followed by the mitral valve (3); there were no reported cases to occur on the tricuspid or pulmonic valves. A total of 11 patients underwent surgical intervention in addition to antibiotics for treatment. Out of three reported deaths, two of them did not undergo valve replacement.

**Table 2:** Review of previously reported cases of endocarditis caused by Legionella. PCR: polymerase chain reaction; M: male; F: female; y: years.

Author	Location	Year	Age/sex	Reason for admission	Valve location/type	Diagnostic testing	Treatment/duration	Prognosis
Moran et al. [3]	USA	2024	53 y/F	Dyspnea, cough	Aortic valve/bioprosthetic	Tissue PCR; postmortem culture	Ciprofloxacin	Death
Teira et al. [4]	Spain	2022	63 y/M	Fever	Aortic valve/bioprosthetic	Tissue PCR	Surgical intervention	Death
Fraz et al. [2]	Norway	2022	66 y/M	Fever, cough, weight loss	Aortic valve/bioprosthetic	Tissue PCR	Levofloxacin + azithromycin for 6 weeks; permanent pacemaker; surgical intervention	Good
Young et al. [5]	USA	2019	80 y/M	Fever	Mitral valve/bioprosthetic	PCR, serology	Levofloxacin for 6 weeks; surgical intervention	Good
Baumgartner et al. [6]	Switzerland	2016	64 y/M	Fever	Aortic valve/native	Serology	Clarithromycin for 6 weeks	Good
Compain et al. [7]	France	2015	58 y/F	Congestive heart failure	Aortic valve/native	PCR	Levofloxacin for 3 weeks; surgical intervention	Good
Thurneysen et al. [8]	Switzerland	2014	70 y/F	Arthritis	Mitral valve/native	Synovial culture	Ciprofloxacin for 3 months	Good
Fukuta et al. [9]	USA	2012	57 y/M	Brain abscess	Mitral valve/mechanical prosthetic	PCR	Levofloxacin for 5 months; surgical intervention	Good
Leggieri et al. [10]	France	2012	73 y/M	Fever, weight loss	Aortic valve/bioprosthetic	PCR, serology	Erythromycin + ciprofloxacin for 6 weeks; surgical intervention	Good
Pearce et al. [11]	USA	2011	68 y/F	Chest pain	Aortic valve/native	Tissue PCR	Moxifloxacin for 6 weeks (refused surgery)	Death
Patel et al. [12]	USA	2005	63 y/M	Fever	Aortic valve/bioprosthetic	Blood culture	Levofloxacin for 36 months, surgical intervention	Good
Massey et al. [13]	United Kingdom	2003	28 y/M	Congestive heart failure	Aortic valve/bioprosthetic	-	Clarithromycin + rifampin (unknown duration), surgical intervention	Good
Chen et al. [14]	USA	1996	33 y/M	Fever	Aortic valve/mechanical prosthetic	-	Erythromycin for 6 months, surgical intervention	Good
Park et al. [15]	USA	1994	65 y/M	Congestive heart failure	Aortic valve/bioprosthetic	Serology	Doxycycline for 6 months, surgical intervention	Good
Littrup et al. [16]	Denmark	1987	38 y/F	Fever	Aortic valve/native	Serology	Erythromycin for 8 weeks	Good
McCabe et al. [17]	USA	1984	60 y/F	Fever	Aortic valve/bioprosthetic	Valve culture, serology	Erythromycin + rifampin for 6 months; surgical intervention	Good

Blood culture-negative infective endocarditis (BCNE) is known to be a challenging condition associated with increased morbidity and mortality. When suspecting this condition, a detailed approach must be taken. This includes obtaining a thorough patient history, testing serum and blood, and appropriate handling of blood cultures, serological testing, and molecular techniques [18]. Causes of BCNE, such as Legionella species, are often overlooked since serologic testing of this bacterium is associated with false positive results, especially if the patient has had prior exposure to this organism [18]. A recurring theme in BCNE caused by Legionella species is that they often present with indistinct clinical findings such as fever, cough, headache, diarrhea, and confusion [19].

In addition to experiencing these symptoms, our patient began to deteriorate following admission to the hospital and developed atrial fibrillation (AF). AF is a common finding in infective endocarditis. However, studies have shown differing opinions on how much it correlates to the clinical outcome of patients with just endocarditis. Studies have demonstrated that patients with a new onset of AF due to infective endocarditis have a higher incidence of in-hospital death, but this does not include BCNE [20]. Documented cases of BCNE due to legionellosis are highly associated with patients who have prosthetic heart valves. However, BCNE due to legionellosis should not be ruled out if the patient does not have prosthetic heart valves because research has identified novel variations in genotypic and phenotypic analyses in Legionella strains to include native valves [11]. This should prompt those suspecting BCNE to use alternative diagnostic tests such as PCR to rule out atypical causes of Legionella rather than relying on blood cultures. Additionally, other causes of BCNE are exposure to antibiotics prior to blood culture due to its ability to sterilize blood [18].

## Conclusions

In conclusion, this case highlights the rare but significant presentation of native valve endocarditis caused by Legionella. While typically associated with pulmonary infections, Legionella can lead to serious extrapulmonary manifestations, including infective endocarditis, even on native valves, which are less commonly involved than prosthetic valves. The rarity of this presentation underscores its clinical importance, as the involvement of native valves by Legionella is exceptionally uncommon in reported cases of infective endocarditis.

The patient’s atypical presentation, including AF and a history of recent travel, underscores the need to consider atypical pathogens like Legionella in the differential diagnosis of common clinical syndromes. Early recognition and appropriate antibiotic therapy are crucial for managing Legionella endocarditis, particularly in cases of BCNE. This case also serves as a reminder of the diagnostic challenges posed by BCNE and highlights the importance of using alternative diagnostic methods, such as PCR, when traditional culture-based techniques fail. Incorporating these considerations into clinical practice is essential for improving the timely diagnosis and treatment of rare pathogens like Legionella in endocarditis, particularly in patients with nontraditional risk factors.
